# Health Tract, No. 257

**Published:** 1866-09

**Authors:** 


HEALTH TRACT, No. 257.

### SUMMER HEALTH.

AUGUST is the most fatal month in towns and cities—October the least.—Nearly twice as many die in August as in December. The deaths in New York during July, August and September are nearly one-third greater than during October, November and December. These proportions most likely hold good else-where: but of these three thousand deaths and sixty thousand cases of sickness besides, more than one half are avoidable, in the estimation of scientific medical men;—avoidable in the main, simply by avoiding the sun from ten to four, and eating and drinking wisely.

All who possibly can should leave the large cities before the first of July, (for then the excessive hot nights begin, and the thermometer stands at ninety degrees, Fareinheit, at sun-rise, in the halls and parlors of our dwellings,) and remain till the first of September. At least three or four weeks in August should be spent in the country by those who can not spare the longer time.—The mountains are better than the sea-shore. One should not choose a river-bank, or bottom-land, or a level country, unless among the pines, because in these situations the seeds of fever and ague are sown, and the person exposed will return to town to have "creeps" or intermittants when cold nights come.

If families, and parties of ladies and gentlemen, were to inaugerate a custom of camping out in the Adriondack mountains or those of Pennsylvania, Virginia or New England for six weeks during the Summer, the advantages over the Springs and the Sea-Side would be incalculable. Young men should travel on foot or on horse-back, and all who can should select boarding-places away from the public thoroughfares, and where they can live as untrameled as possible by dress, fashions, and formalities of every description. Still, observant people must know that there is more real enjoyment and comfort in one's own house in the city. The freedom of the whole building, night and day; your own hours of rising and retiring; your baths; your *dishablle*;—these are indispensible to real comfort any where in mid-summer. But the great multitude must stay at home the year round, and it can be done in good health if, other things being equal, a wise system of eating and drinking were adhered to, on the following succinct principles: Take coffee, tea, cold water, lemonade and other acids, and ice-cream. It is believed by the best French physiological experimenters and observers, that all acids, especially in Summer, promote the secretion of bile, provent fevers, and keep the system free; hence the advantage of fruits, berries, kole slaw, salads, pickles, sour milk, and the like, as warm weather approaches. Sweet milk, ale, beer and porter, all tend to create bile, to constipate, to induce head-ache, cold feet, neuralgia, and want of appetite, in Summer. The whole body is weak and indisposed to effort in warm weather. The stomack is in the same relaxed condition, and to impose on it full meals, and to urge it to take fuller ones by tonics, stimulates, and tempting tables, is irrational and suicidical, and is the immediate cause of one half the sickness and deaths of warm weather, for they are avoidable by the following system of diet:

Eat but three times a day and nothing between meals. Breakfest: coffee, tea, or cold water; cold bread and butter and a saucer or two of berries in their natural state, ripe, fresh, and perfect. Supper, same except no berries. Dinner, lemonade or cold water, bread and butt er with tomatoes, or any other one vegetable. Meats one day, soups another; melons and berries as above, for dessert. Bread and butter and cold water alone would sustain life and give vigorous health for the Summer months. Any family who will diet as above for one week in Summer time, avoiding ordinary exposures, will find an exemption from "unpleasant" symptoms which will convinco them at once of the value of such a system of living.—with a lightness of spirits, a joyousness of mind and a mirthfulness of temper, which is a real luxury to think of, in comparison to the weariness, dulness, want of appetite, suffering from heat, wakeful nights, unre reshed mornings, insufferable ennui, and intense longing for excitement and exciting drinks,—which afflict those who sit at luxurious tables all Summer.

